# Instrument-based tests for measuring anterior chamber cells in uveitis: a systematic review protocol

**DOI:** 10.1186/s13643-019-0946-3

**Published:** 2019-01-22

**Authors:** Xiaoxuan Liu, Ameenat L. Solebo, Pearse A. Keane, David J. Moore, Alastair K. Denniston

**Affiliations:** 1 Ophthalmology Department, University Hospitals Birmingham NHS Foundation Trust, Birmingham, UK; 2 Academic Unit of Ophthalmology, Institute of Inflammation and Ageing, University of Birmingham, College of Medical and Dental Sciences, Birmingham, UK; 3 Institute of Child Health, University College London, London, UK; 4 NIHR Biomedical Research Centre for Ophthalmology, Moorfields Eye Hospital NHS Foundation Trust and UCL Institute of Ophthalmology, London, UK; 5 Institute of Applied Health Research, College of Medical and Dental Sciences, University of Birmingham, Birmingham, UK; 6 Centre for Rare Diseases, Birmingham Health Partners, Institute of Translational Medicine, Birmingham, UK

**Keywords:** Systematic review, Uveitis, Anterior chamber cells, Aqueous humour, Monitoring test, Diagnostic test, Optical coherence tomography, Laser flare-cell photometry

## Abstract

**Background:**

Uveitis describes a group of inflammatory conditions affecting the eye. The ability to monitor inflammatory changes in anterior uveitis is crucial in clinical practice for making treatment decisions and in clinical trials for testing therapeutic agents. The current standard for quantifying anterior segment inflammation is clinical slit-lamp examination findings classified using the Standardisation of Uveitis Nomenclature (SUN) grading system. Such clinical grading systems rely on a subjective estimation using the slit lamp and are often non-linear and non-continuous scales, with large increases in cell count between each grade. Novel instrument-based technologies have emerged over the last few decades, which can provide objective and quantifiable measurements. This review will evaluate the reliability of such technologies and their level of agreement with anterior chamber (AC) cell count using clinical slit-lamp examination.

**Methods:**

Standard systematic review methodology will be used to identify, select and extract data from studies that report the use of any instrument-based technology in the assessment of AC cells. Searches will be conducted through bibliographic databases (MEDLINE, EMBASE and Cochrane Library), clinical trial registries and the grey literature. No restrictions will be placed on language or year of publication. The outcomes of interest are the correlation of index test measurements of AC cells with clinical grading systems using slit-lamp examination and the reliability of each index test identified. Quality assessment will be undertaken using QUADAS2. Degree of correlation between the index and reference test measures will be pooled and meta-analysed if appropriate.

**Discussion:**

A number of instrument-based tools are available for measuring AC cells. This review will evaluate the technologies available and measure the level of correlation of these alternative methods with clinical grading systems as well as their performance in reliability and repeatability. The findings of this review will identify those objective, instrument-based technologies which show good utility for measuring AC cells in a quantifiable way and which warrant further exploration for their sensitivity and reliability over the current standard.

**Systematic review registration:**

PROSPERO CRD42017084156 (Liu X, Moore DJ, Denniston AK). Instrument-based tests for measuring anterior chamber (AC) cells in uveitis: a systematic review. 2017). Study screening stage is complete. Data extraction stage has not yet commenced.

## Background

Uveitis is a group of inflammatory conditions affecting the eye. It is a major cause of blindness globally, with an estimated prevalence of 38 to 114.5 per 100,000 population [1–3]. Uveitis can occur in any age, but it has a tendency to affect the working-age group, thus having a high socio-economic impact [1].

The Standardisation of Uveitis Nomenclature (SUN) Working Group classifies uveitis based on the anatomical focus of inflammation: anterior (anterior chamber), intermediate (vitreous), posterior (retina and/or choroid) and panuveitis (all anatomical parts) [4]. This systematic review focuses on inflammation in the anterior chamber, where inflammation causes a disruption to the normal blood-aqueous barrier, resulting in leakage of cells into the aqueous humour. These cells can be observed as floating particles in the anterior chamber (AC). An increase or decrease in the number of AC cells can be indicative of improving or worsening disease and are critical in identifying active inflammation and rationalising treatment decisions [4].

### Clinical examination of AC cells

The current standard measurement as defined by the SUN grading system is clinical examination by slit-lamp biomicroscopy, whereby a clinician aims a 1 × 1-mm slit beam through the anterior chamber and counts the number of cells visible in the lit area [4]. The cell count is placed into one of six grades in the SUN grading system (Table 1). Prior to the SUN grading system, a number of alternative systems existed which attempt to quantify cells in the same way; however, the SUN grading system is now the accepted standard for clinical practice and for clinical trials [5–9].

**Table 1:** The Standardisation of Uveitis Nomenclature (SUN) working group grading scheme for anterior chamber cells

Grade	Cells in field
0	< 1
0.5 +	1–5
1 +	6–15
2 +	16–25
3 +	26–50
4 +	> 50

Standardisation of uveitis nomenclature for reporting clinical data. Results of the First International Workshop. [4]

### Optical coherence tomography

In the last few decades, novel ophthalmic imaging techniques such as optical coherence tomography (OCT) have become widely adopted and provide new ways of quantifying disease through instrument-based measurements. OCT is fast, non-invasive and provides a quantifiable measure of ocular structure that is more sensitive and reliable than clinical estimates. Anterior segment optical coherence tomography (AS-OCT) can provide cross-sectional imaging of the AC and is able to identify cells in the anterior chamber as hyper-reflective dots. Several studies have suggested that AC cell count on OCT correlates with clinical grading [10, 11]. Additionally, the number of dots/cells in a given volume of aqueous humour can be counted manually or with automated software [12].

### Laser flare and cell photometry

The laser flare meter was introduced in 1988 for quantification of anterior chamber protein and cells [13]. It is a fast and non-invasive technique which measures the amount of light scatter from particles as a laser beam is projected into the anterior chamber. The amount of back-scattered light is proportional to the concentration and size of proteins and cells in the aqueous humour.

Laser flare-cell photometry is primarily used in the estimation of AC flare (the hazy appearance given to the aqueous fluid by inflammatory proteinaceous leakage); however, certain models can be used for counting AC cells. Counting of cells by laser photometry is reported to be less accurate than its use in measuring flare, particularly in the extreme ends of the spectrum [14].

### Purpose

Although we are aware of two main instrument-based techniques for quantifying AC cells, it is possible that more technologies, newer generations of the same technologies or the same technologies accompanied by newer acquisition techniques and software automation are available. The aim of this systematic review is to investigate all instrument-based methods for quantifying AC cells and evaluate the correlation of these measures with clinical grading using slit-lamp examination in patients with uveitis. Where reported, we will also compare the level of reliability and repeatability for these methods to determine which yields the most reliable results. For the purposes of this review, we will refer to all instrument-based methods as ‘index tests’.

## Aim

To investigate which instrument-based technologies for measuring anterior chamber cells in uveitis are available and assess their level of validation.

The following questions are proposed:

- Which non-invasive instrument-based tests (index tests) have the potential to measure anterior chamber cells in uveitis?
- What is the level of agreement between each index test and grading by clinical examination?
- What is the reliability and repeatability of each index test?

## Methods

### Protocol

This protocol is designed as per guidelines of Preferred Reporting Items for Systematic Review and Meta-Analysis Protocol (PRISMA-P) [15]. The systematic review will be reported in accordance to the PRISMA guidelines [16].

### Systematic review registration

Our protocol has been registered on PROSPERO (CRD42017084156) [17].

## Searches

### Databases

The relevant data for this review is likely to come from studies assessing a variety of quantitative research questions in uveitis; therefore, our search strategy will reflect the pathological finding of interest ‘anterior chamber cells’ and the disease context ‘uveitis’. No search terms will be applied for the ‘technologies/tests’ to maximise sensitivity of the search. For bibliographic databases, free text and index terms (where available) will be combined for each search element. A sample search strategy for MEDLINE can be found in the Appendix. We will search:

- Bibliographic databases of published studies
  - MEDLINE (Ovid), 1946–present
  - Embase (Ovid), 1947–present
  - The Cochrane library, database inception–present
  - Centre for Reviews and Dissemination database (Health Technology Assessments and the Database of Abstracts and Reviews of Effects), database inception - present
- Registers of clinical trials
  - Clinicaltrials.gov (www.clinicaltrials.gov)
  - WHO International Clinical Trials Registry Platform (ICTRP portal) (www.who.int/ictrp)
- Abstract and conference proceedings
  - British library Ethos
  - ProQuest (www.proquest.com)
- Dissertations and theses
  - British Library’s ZETOC
  - Conference Proceedings Citation Index (Web of Science)
- Grey literature
  - OpenGrey (www.opengrey.eu)

No restrictions will be placed on year or language of publication. The literature search results will be entered onto EndNote × 8.2 (Clarivate Analytics, Philadelphia, PA) to facilitate removal of duplicates, study selection, recording decisions and references. References to other works will be considered for inclusion.

## Selection criteria

### Participants/population

The populations of interest are those with evidence of anterior chamber cells and/or a diagnosis of uveitis, irrespective of active or inactive inflammation. There will be no restrictions on age, gender, ethnicity, or underlying aetiology or anatomical subtype. Studies with only healthy participants and studies on animals will not be included.

### Index test

Included studies should describe one or more instrument-based methods for counting the number of anterior chamber (AC) cells.

### Comparator

Included studies should use a clinical grading system (such as the SUN grading system) as a comparator.

### Primary outcome

The primary outcome is the correlation between index tests and the clinical grading system; the reported outcome should be a correlation coefficient for the two measures. Studies which do not report a correlation coefficient but report matched measurements for the index and reference tests can be included, and the correlation coefficient extrapolated during analysis.

### Additional outcome

To assess the reliability of a test, inclusion of a comparator is not required. The outcome of interest is intra/inter-observer reliability and repeatability of an index test in the same population as above.

### Type of study

There will be no restrictions on study design; however, evaluation of correlation between index test and clinical grading using slit-lamp examination requires both tests to be done in a cross-sectional manner. Only those studies where measurements are taken within a reasonable time point (within 24 h of each other) will be included.

Case reports involving only one subject, commentaries, opinion articles and pictorial articles will not be included.

## Selection process

Titles and abstracts of records returned by the searches will be screened for relevance to the review, to remove obviously irrelevant studies Two independent reviewers will carry out quality assessment and reach consensus by discussion or referral to a third reviewer.

Full text of potentially relevant articles will be retrieved and assessed for inclusion in the review against the full selection criteria.

## Data extraction

Data will be extracted from the included studies using a standardised data extraction form in Microsoft Excel (Microsoft, Washington, UK). The extraction process will be carried out by two independent reviewers with referral to a third reviewer if necessary. Information to be extracted from all studies include the following:

- Study characteristics
  - Title, authors, publication year, journal and language
  - Sample size
  - Study design
  - Index test used
    1. Manufacturer and model (including resolution and scan speed)
    2. Image acquisition settings
    3. Area, volume and position scanned in the AC
    4. Software for image analysis and level of automation (manual, semi-automated or fully automated)
  - Clinical grading system measurements
    1. Observer (same observer or different observers)
    2. Description of grading system used, including the name of the system, number of grades and number of cells in each grade
    3. Slit lamp settings such as area and brightness of illumination
- Patients’ characteristics
  - Age, gender and ethnicity
  - Underlying aetiologies (type of uveitis, anatomical subtype, aetiological classification)
  - Active or inactive disease
  - If the study involves a therapeutic intervention: treatment details (indication, drug, dosage, route, subject pre/post-treatment status and length of follow-up)
- Outcomes and findings
  - Data will be extracted in preparation for two separate analyses depending on the data reported:
    1. *Evaluation of correlation between index tests and a clinical grading system:* Studies which include AC cell measurement by both index test and a clinical grading system in the same population will be used to evaluate the level of correlation between the two tests. The correlation coefficient reported will be directly extracted. If no correlation coefficient is reported, we will extract index and reference test measurements, providing they are matched, and calculate the correlation coefficient. If the two measurements are not matched, we will contact the authors for matched measurements.
    2. *Evaluation of reliability and repeatability of an index test:* Studies which report intra- and inter-observer reliability for a study will be analysed separately for assessment of repeatability. The reported kappa values will be extracted for intra-observer reliability and inter-observer reliability.
- Cross-sectional measurements may be nested within longitudinal studies whose primary aims are not to compare performance or correlation between two tests. In this situation, measurements at each time point should be extracted and analysed as individual cross-sectional comparisons
- Results of sub-analyses and sensitivity testing for uveitis subtype (aetiological and anatomical) and disease activity (active versus inactive disease)

## Quality assessment

Quality assessment of all included studies will be based on elements from the Quality Assessment of Diagnostic Accuracy Studies tool (QUADAS2) [18]. Assessment will be carried out at the study level. Two independent reviewers will carry out quality assessment and refer to a third reviewer if needed.

It is anticipated that a small number of studies will meet the inclusion criteria, with many of which being early proof-of-concept studies with a high risk of bias. Therefore, the risk of bias assessment and quality of evidence will be reported but will not influence data synthesis.

For studies assessing the correlation between an index and reference test, the following four risk of bias domains will be rated as low, high or unclear [18]:

1. Selection of participants
  - Selection of participants with different degrees of disease severity should be justified in the context of assessing the full spectrum of disease, as opposed to any prior knowledge of how disease severity may affect test performance. As severe inflammation (i.e. grade 4+) is less prevalent than mild inflammation (i.e. + 0.5), a random selection of participants would invariably result in more participants with milder disease than participants with severe disease.
  - Exclusion of participants should be justified in the context of interference with index test measurement (i.e. corneal opacities may prevent visualisation of AC structures behind it, or the presence of AC pigment may appear like AC cells but is caused by a different pathological process).
2. Index tests
  - We will consider whether index test acquisition and analysis parameters were determined a priori and consistent for all participants in the study.
  - If analysis of index tests were done manually (i.e. manual counting of AC cells in an image), we will consider whether the observer was blinded to the clinical AC cell grading, and vice versa.
3. Reference test
  - We will consider whether assessment using the clinical grading system in each study was standardised, i.e. performed by the same clinician, consistent slit-lamp settings such as brightness of illumination and ambient settings such as room lighting.
4. Flow and timing
  - Order of tests: The order of tests does not affect the interpretability of results if the observer of one test is blinded to the results of the other test.

For studies assessing the reliability of a single index test, the same assessment should be done for selection of participants and index tests as above. Additional considerations should be given for:

1. Intra-observer reliability studies: the conditions under which index test repeatability was performed should be reported and kept consistent (i.e. room lighting, slit lamp beam intensity).
2. Inter-observer reliability studies: as well as the points relating to intra-observer reliability, any differences between observers (such as seniority and experience) should be reported and accounted for.

## Data synthesis

Data synthesis will be divided into analysis for the two outcomes: correlation with reference test and reliability of index test. For each outcome, a narrative synthesis of tabulated evidence will be conducted and supported with a meta-analysis where possible.

1. Evaluation of correlation between index and reference test
  - These studies will be grouped by type of technology (i.e. OCT, laser flare cell photometry).
    For each type of technology, we are expecting small numbers of studies; therefore, we will analyse different platforms, generations or manufacturers of the same technology together.
  - Studies will then be grouped by comparator (i.e. different clinical grading systems).
  - If the data allows, correlation coefficients between each index test versus reference test will be compared and pooled for meta-analysis.
2. Evaluation of reliability and repeatability of an index test
  - These studies will also be grouped by type of technology, as described above.
  - Intra-observer and inter-observer reliability will be analysed separately for each technology. If the data allows, reliability measure (i.e. Kappa values) will be compared and pooled for meta-analysis.

Assessment of clinical and methodological homogeneity will determine whether studies are sufficiently similar to allow for appropriate data pooling by meta-analysis. Heterogeneity across studies in each meta-analysis will be quantified using the *I*^*2*^ statistic. Irrespective of the ability to appropriately undertake any meta-analyses, data will be reported narratively across all studies for each grouping.

We will perform sensitivity analyses if there is significant heterogeneity between studies. If data permits, we will consider subgroup analyses for population groups (i.e. age, gender and ethnicity), anatomical subtype of uveitis and aetiological subtype. It is anticipated that only a small number of studies will be relevant to the inclusion criteria, and this may limit our ability to carry out meaningful subgroup analyses.

## Discussion

The assessment of uveitis is complex: firstly, because uveitis describes a heterogeneous group of diseases with significant variations between different anatomical and disease-specific subtypes, and secondly, because many clinical measures in ophthalmology are based on visual function (namely visual acuity), which are subjective to the patient and do not always reflect active inflammation. Treatment for anterior uveitis, namely topical steroids, carry side effects such as increased risk of glaucoma and accelerated onset of cataract. The ability to reliably measure ocular inflammation in uveitis is important for rationalising treatment in clinical practice and assessing disease outcome in clinical trials.

Whilst the consensus Standardisation of Uveitis Nomenclature meeting of 2005 was a significant effort towards defining a systematic method in assessing uveitic inflammation, it is recognised that its reliance on clinical examination remains subjective, unreliable and poorly sensitive. A number of factors can affect the examiner’s ability to see AC cells including the slit lamp optics, the degree of illumination, and the observer’s skills [19]. The problem exists that clinical grading through examination is error-prone and is therefore susceptible to intra-observer and inter-observer variability [19, 20]. Clinical grading systems also typically classify levels of severity into stepwise grades, in a non-continuous and non-linear fashion, with a wider range of cell counts in the higher grading groups (i.e. 26–50 cells in field for grade 3+ and > 50 cells in field for 4+) than lower grading groups [4]. According to the SUN criteria, “improved activity”, or a decrease in inflammation, requires a two-step improvement or resolution to grade 0. However, a much larger reduction of AC cell count is required in higher grades of inflammation (i.e. 4+ to 2+ requires 50+ cells decreasing to 16–25 cells) than lower grades (i.e. 2+ to 0.5+ requires 16–25 cells decreasing to < 1 cells). This non-linear grading scale may not be able to detect small changes, especially within higher grades of inflammation, allowing potentially clinically meaningful changes to go undetected. In clinical trials, this could result in new therapies being deemed as failure, despite having a clinically significant improvement.

Instrument-based technologies such as the laser flare-cell photometer and OCT have shown potential for assessing anterior chamber cells in several studies. These instrument-based measures are less operator-dependent and carry advantages such as objectivity. However, their performance in these domains has not been compared in a systematic way. The field of ophthalmic imaging is continuing to expand rapidly; therefore, it is timely to undertake a systematic review to examine the evidence for instrument-based measures of intraocular inflammation.

This systematic review will explore the range of technologies available for measuring AC cells to estimate their level of reliability and correlation with clinical grading systems. The findings from this review would contribute to the process of validating instrument-based methods for guiding treatment decisions in clinical care and for measuring outcome in uveitis clinical trials.

## Appendix

**Table 2:** MEDLINE sample search strategy

1	Exp Uveitis/
2	Uveiti*. Ti, ab.
3	1 or 2
4	Anterior chamber. Ti, ab.
5	Aqueous humour. Ti, ab.
6	Aqueous humor. Ti, ab.
7	4 or 5 or 6
8	Cell*. Ti, ab.
9	3 and 7 and 8

## Abbreviations

AC: Anterior chamber
MEDLINE: Medical Literature Analysis and Retrieval System Online
OCT: Optical Coherence Tomography
PRISMA: Preferred Reporting Items for Systematic Reviews and Meta-Analysis
QUADAS2: Quality Assessment of Diagnostic Accuracy Studies
SUN: Standardisation of Uveitis Nomenclature
